# Draft Genome Sequence of *Burkholderia cenocepacia* Strain CEIB S5-2, a Methyl Parathion- and *p*-Nitrophenol-Degrading Bacterium, Isolated from Agricultural Soils in Morelos, Mexico

**DOI:** 10.1128/genomeA.00220-16

**Published:** 2016-04-28

**Authors:** Fernando Martínez-Ocampo, Maikel Gilberto Fernández López, Luis Fernando Lozano-Aguirre Beltrán, Elida Carolina Popoca-Ursino, M. Laura Ortiz-Hernández, Enrique Sánchez-Salinas, Fernando Ramos Quintana, Miguel A. Villalobos-López, Edgar Dantán-González

**Affiliations:** a Laboratorio de Investigaciones Ambientales, Centro de Investigación en Biotecnología, Universidad Autónoma del Estado de Morelos, Cuernavaca, Morelos, México; b Programa de Genómica Evolutiva, Centro de Ciencias Genómicas, Universidad Nacional Autónoma de México, Cuernavaca, Morelos, México; c Centro de Investigación en Biotecnología Aplicada, Instituto Politécnico Nacional, Tepetitla, Tlaxcala, México

## Abstract

*Burkholderia cenocepacia* is an opportunistic pathogen that belongs to *Burkholderia cepacia* complex (BCC). *Burkholderia cenocepacia* strain CEIB S5-2 was isolated from agricultural soils in Morelos, Mexico, and previously has shown its abilities for bioremediation. In this study, we report the draft genome sequence of *Burkholderia cenocepacia* strain CEIB S5-2.

## GENOME ANNOUNCEMENT

The *Burkholderia cepacia* complex (BCC) is a group of Gram-negative bacteria composed of at least 18 different species, including *Burkholderia cenocepacia*, which is common in the environment as a free form or associated with plants (1) and is also an opportunistic pathogen in patients with cystic fibrosis (2). The *B. cenocepacia* strain CEIB S5-2, as well as other strains of this genus (*B. zhejiangensis* strain CEIB S4-3 [3] and *B. cenocepacia* strain CEIB S5-1 [4]), was isolated from agricultural soils in Tepoztlán, Morelos, Mexico (5). Importantly, *B. cenocepacia* CEIB S5-2 has the capability to hydrolyze methyl parathion (MP) and use it as a carbon source, and to degrade completely *p*-nitrophenol (PNP) in 21 h (3). Here, we present the draft genome sequence of the organophosphorus pesticide-degrading strain *B. cenocepacia* CEIB S5-2.

Genomic DNA was obtained using the Ultra-Clean Microbial DNA isolation kit (Mo Bio Laboratories), and 5 µg of genomic DNA was sequenced in the HiSeq 2000 system (Illumina). We obtained a random data set of 1,842,982 paired-end reads with lengths of 300 bp. Quality-based trimming was performed with a DynamicTrim (SolexaQA++) Perl script, and genome assembly was accomplished using SPAdes version 3.1.1. The draft genome has 109 contigs with a calculated total length of 8,976,170 bp, an *N*_50_ contig size of 201,068 bp, a G+C content of 65.68%, and ~62× coverage. We identified the 16S rRNA gene of *B. cenocepacia* CEIB S5-2 using RNAmmer version 1.2 (http://www.cbs.dtu.dk/services/RNAmmer/) and comarping the strain with 67 16S rRNA gene sequences of genus *Burkholderia* and two 16S rRNA genes of genus *Ralstonia* (outgroup). All sequences were aligned with the MUSCLE server (http://phylogeny.lirmm.fr/phylo_cgi/one_task.cgi?task_type=muscle), and a phylogenetic analysis was performed with MEGA version 6.1 with the neighbor-joining algorithm, using 1,000 replicates for bootstrapping. Phylogenetic analysis reveals that *Burkholderia cenocepacia* CEIB S5-2 is closely related to *Burkholderia cenocepacia* spp*.* The 16S rRNA gene of *B. cenocepacia* CEIB S5-2 has a length of 1,521 bp and has 99% identity and 100% alignment coverage with *B. cenocepacia* CEIB S5-1 (5), *B. cenocepacia* H111 (6), and *B. cenocepacia* 869T2 (7). We did not identify pathogenicity islands of *B. cenocepacia* CEIB S5-2 using the Pathogenicity Island Database (PAIDB) (http://paidb.sybirg.re.kr/about_paidb.php) server.

The 109 contigs were analyzed with the RAST server (http://rast.nmpdr.org), identifying 8,142 open reading frames and 8,034 coding sequences. We identified a PNP catabolic gene cluster: *pnpABA’E1E2FDC*, positive ranging from 79 to 100% with PnpABE1E2FDC proteins from *Burkholderia* sp. SJ98 (8, 9) using tBlastn program. While *Burkholderia zhejiangensis* CEIB S4-3 and *Burkholderia cenocepacia* CEIB S5-1 and S5-2 have the capability to completely degrade PNP, the strain S4-3 has two catabolic gene clusters (*pnpABA’E1E2FDC* and *pnpE1E2FDC*) (4), the strain S5-2 has one catabolic gene cluster (*pnpABA’E1E2FDC*) and the strain S5-1 does not has a catabolic gene cluster reported (5). Furthermore, the total genome size of *Burkholderia cenocepacia* CEIB S5-2 is 1,309,327 and 920,672 bp larger than that of strains S4-3 (8,976,170 bp and 154 contigs) (4) and S5-1 (7,666,843 bp and 177 contigs) (5), respectively.

### Nucleotide sequence accession numbers.

This whole-genome shotgun project has been deposited at DDBJ/EMBL/GenBank under the accession number LNCR00000000. The version described in this paper is the first version, LNCR01000000.
